# Huge bilateral polycystic kidneys with suspicion of malignancy, recurrent bleeding in cysts, and acute abdomen

**DOI:** 10.3332/ecancer.2012.267

**Published:** 2012-09-04

**Authors:** TAN Bhatty, MS Moazin, R Haque

**Affiliations:** Division of Urology, Department of Surgical Specialities, King Fahad Medical City, Riyadh, Saudi Arabia

**Keywords:** *polycystic*, *kidney*, *nephrectomy*, *malignancy*

## Abstract

We present a case of huge bilateral polycystic kidneys, with suspicion of malignancy and repeated admissions with acute abdomen, secondary to bleeding in cysts, and anaemia, requiring affected side nephrectomy.

**Key message:**

Autosomal dominant polycystic kidney disease (ADPKD) mostly ends up with end stage renal disease (ESRD), requiring haemodialysis, with increased risk of malignancy and enlargement of kidneys with its associated complications, mostly dealt with conservatively, except malignancy, which requires nephrectomy.

## Introduction

Autosomal dominant polycystic kidney disease (ADPKD) is one of the most common hereditary kidney disorders, affecting 8 to 10% of patients with end stage renal disease (ESRD) in the United States and Europe [1]. Most of the complications of ADPKD can be managed by conservative means and nephrectomy is rarely required, as was in our case.

## Case report

We report on a 42-year-old Saudi female, known to have ADPKD with ESRD, for which she was on regular haemodialysis. She had very frequent emergency admissions with severe left half abdominal pain, gross haematuria, vomiting, and anaemia, requiring repeated blood transfusions. Physical examination revealed a distended abdomen completely filled by huge bilateral polycystic kidneys. Bowel sounds were normal. Ultrasound and non-contrast CT revealed bilateral huge polycystic kidneys, with a right kidney size of 362 × 135 mm and a left kidney size of 311 × 144 mm, with blood clots in a large left renal cyst measuring 173 × 117 mm in size. Also noted was a nodular lesion of 30.2 × 20.4 mm in the lower pole of the left kidney, with suspicion of malignancy.

**Figure 1:**
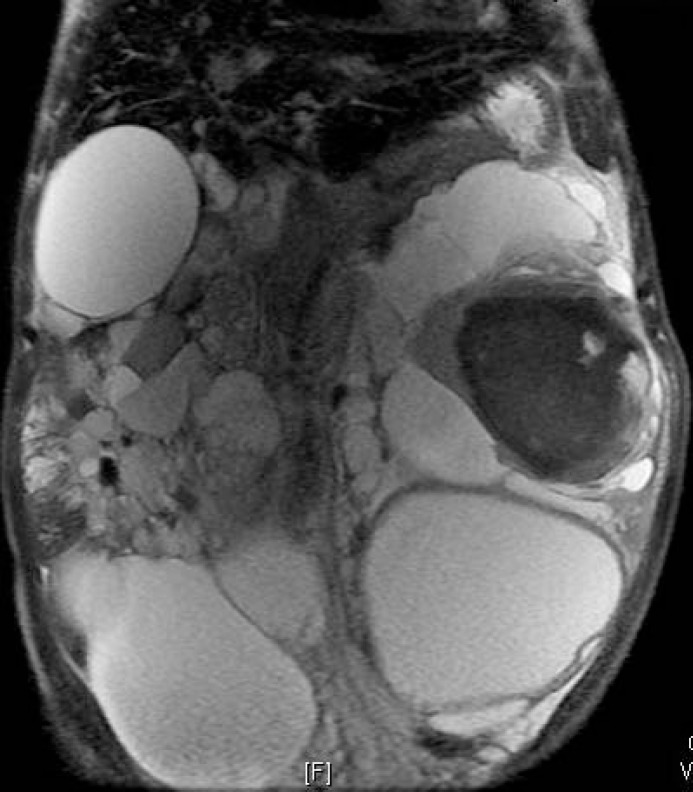
MRI showing bilateral polycystic kidneys with bleeding in left renal cyst.

**Figure 2:**
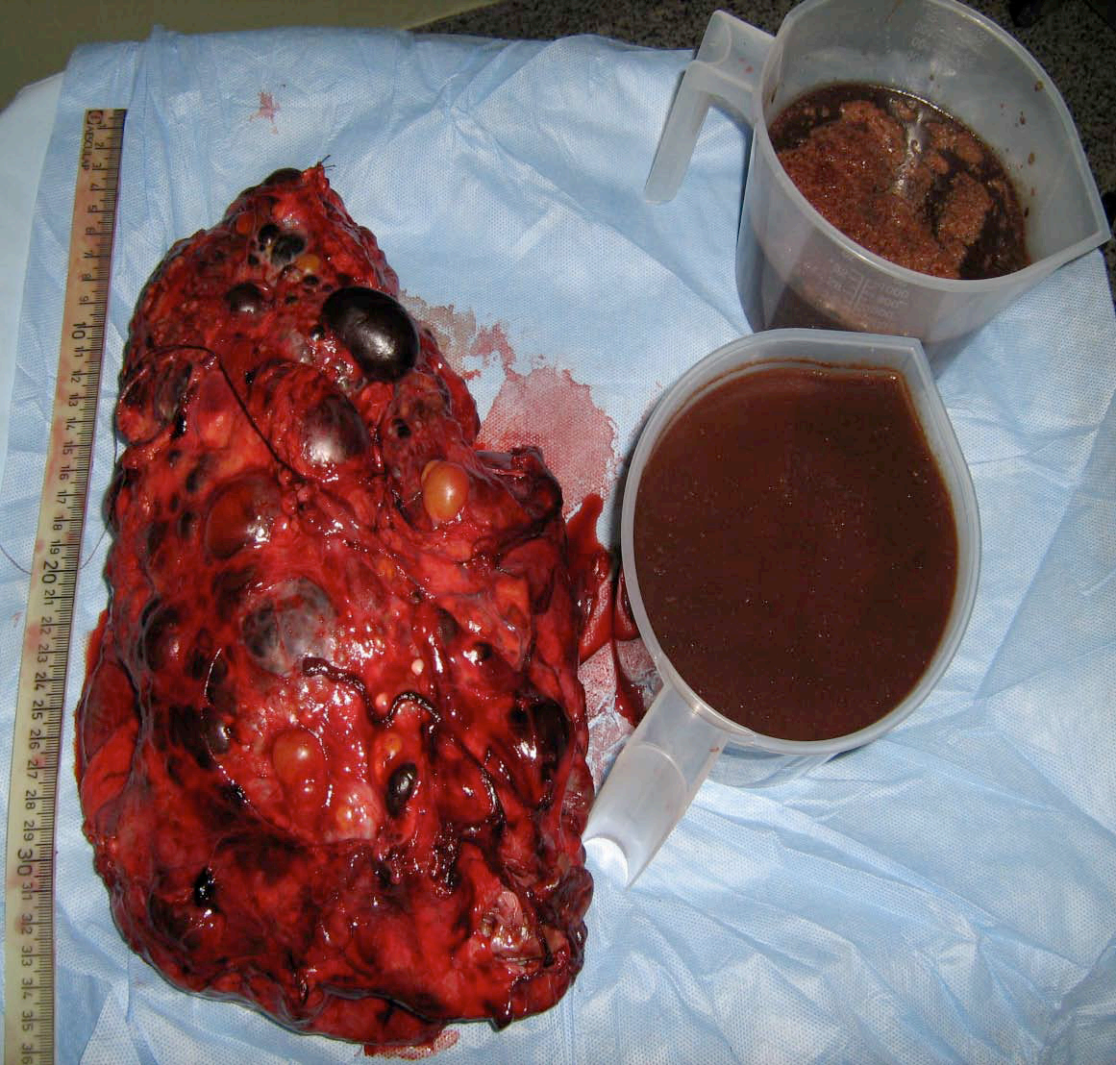
Left nephrectomy specimen weighing 3.5 kg along with 1,800 ml of aspirated fluid.

An MRI with contrast could not differentiate between a recent haemorrhage and tumour (Figure 1). Hence, the patient underwent a left nephrectomy via left sub-costal transperitoneal approach. The intraperitoneal contents were normal. The left nephrectomy was done, and the excised specimen weighed 3.5 kg, in addition to 1,800 ml of black tarry fluid aspirated during the surgery (Figure 2).

Post-nephrectomy, lavage was done with heparin 500 U in 1,000 ml of normal saline, before wound closure. The surgery and post-operative period were uneventful. The patient had total relief of her symptoms. The biopsy was reported as a polycystic kidney with haemorrhages and micro abscesses, but no evidence of malignancy.

## Discussion

This case is being reported because ADPKD with ESRD has increased risk of renal cell carcinoma (RCC). However, risk of RCC in ADPKD is controversial, as the tumour is difficult to detect because of renal distortion, with reported incidence of about 2–12% [2]. In a study of 79 ADPKD patients with ESRD, who had 89 nephrectomies for suspicion of malignancy, pre-kidney transplant or benign complications (pain, haemorrhage, and recurrent infections). Of these 89 nephrectomies 11 had RCC, while 5 of these 11 RCC cases had nephrectomy on suspicion of malignancy. Four (80%) of these five cases had cancer which was clear cell carcinoma in 58.3% and tubulopapillary carcinoma in 41.7%. All were pT1 (1997 TNM Classification) with Fuhrman grades 1 and 2. The mean tumour size was 18.4 mm (range 1–30 mm). There was high-RCC prevalence rate of 8.3% in ADPKD which increased to 12% after 1 year of dialysis [3]. In another study of 260 pre-transplant nephrectomies in ESRD, a high-RCC prevalence rate was revealed of 4.2% since ESRD associated with acquired cystic kidney disease is a well-known risk factor for RCC [4]. Otherwise RCC prevalence has been reported as 0.9% in an autopsy study [5]. Nephrectomy is, however, avoided for benign complications, as retained native kidneys have useful contributions in potassium excretion, prevention of acidosis, Vitamin D3, and erythropoietin production. Most ADPKD cases on haemodialysis develop renal enlargement with risk of bleeding in cysts, which can be managed by angioembolisation [6.]. Giant bilateral polycystic kidneys can also result in intestinal obstruction with acute abdomen, requiring nephrectomy [7].

There is an association between polycystic kidney cyst progression secondary to activation of mammalian target of rapamycin (mTOR) pathway, promoting renal tubular cell proliferation with tumour formation and angiogenesis. Based on this fact, there are ongoing clinical trials evaluating the role of mTOR inhibitor drugs on polycystic kidney cysts and solid kidney tumours regression [8].

## Conclusions

A high level of RCC suspicion is desired in the follow up of ADPKD, requiring nephrectomy. However, other benign complications of ADPKD can be managed mostly by conservative means and rarely require nephrectomy.
